# Peripheral Biomarkers of Lipid Dysregulation and Inflammation in Anxiety‐Related Risk for Alzheimer’s Disease and Related Dementias

**DOI:** 10.1002/alz70861_108071

**Published:** 2025-12-23

**Authors:** Ana Paula Costa, Helmet T. Karim, Marissa F Farinas, Xuemei Zeng, Meryl A Butters, Stacy L. Gelhaus, Thomas K Karikari, Carmen Andreescu

**Affiliations:** ^1^ University of Pittsburgh, Pittsburgh, PA USA; ^2^ University of Pittsburgh School of Medicine, Pittsburgh, PA USA

## Abstract

**Background:**

Anxiety and its phenotypes are risk factors for several major diseases of aging including cardiovascular and autoimmune diseases, as well as Alzheimer’s Disease and related dementias (ADRD). However, little is known about the mechanisms underlying the association between anxiety and ADRD risk. Leveraging lipidomics and proteomics allows investigation of neurobiological markers contributing to ADRD risk among individuals with specific anxiety phenotypes (rumination, global anxiety, and worry; RAW).

**Method:**

We investigated the relationship between RAW and peripheral biomarkers of lipid dysregulation, inflammation and ADRD. Plasma from 42 participants was collected at baseline [19 high‐RAW (PSWQ≥55 and/or RSQ>50 and/or HARS≥17) and 23 low‐RAW]. Cross‐validated Elastic Net regression and traditional multiple linear regression were applied to investigate associations between NULISAseq CNS Disease Panel 120, untargeted‐lipidomics, and clinical outcomes, and their interaction with APOE4.

**Result:**

Our analysis included data from 42 participants (54.8% females), with a mean age of 64.2 years. We identified a subset of inflammatory (contactin‐2, TAFA5, IL‐5, IL‐9, CCL11, S100A2) and endothelial damage (SAA1, VCAM1) markers significantly associated with worry and rumination in APOE4 carriers, but not with global anxiety. Additionally, lipidomic profiling in plasma revealed multiple differential lipids that were substrates (phosphatidylcholine‐PC, phosphatidylethanolamine‐PE; *p* <0.05) and products (LysoPC‐LPC, LysoPE‐LPE; *p* <0.05) in the Lands Cycle for acyl‐chain remodeling associated with RAW, adjusting for sex and age. Given the smaller sample, we focus on effect sizes and report ß>0.2.

**Conclusion:**

Our results highlight a coordinated plasma lipid profile shift (decreased PC, PE; increased LPC, LPE) in high‐RAW, suggesting disrupted membrane integrity and heightened neuroinflammation, consistent with changes observed in preclinical AD. Additionally, high‐RAW APOE4 carriers showed alterations in markers of inflammation and endothelial damage, suggesting that chronic inflammation may contribute to early vascular dysregulation and serve as a biological pathway linking anxiety‐related phenotypes to ADRD risk. Further research is needed to clarify these biological pathways and explore targeted prevention strategies.

